# Sulfonated Poly(ether ether ketone) Doped with Ammonium Ionic Liquids and Nano-Silicon Dioxide for Polymer Electrolyte Membranes

**DOI:** 10.3390/polym11010007

**Published:** 2018-12-21

**Authors:** Shuguo Qu, Minhui Li, Chenchen Zhang, Yuanyuan Sun, Jihai Duan, Weiwen Wang, Jianlong Li, Xiaojin Li

**Affiliations:** 1 Key Laboratory Reactions and Isolations of Muti-phases Liquid of Shandong Province, College of Chemical Engineering, Qingdao University of Science & Technology, Qingdao 266042, Shandong, China; lime0103@163.com (M.L.); 17864213202@163.com (C.Z.); m15969627512@163.com (Y.S.); duanjihai@qust.edu.cn (J.D.); wangweiwenqd@163.com (W.W.); ljlong321@163.com (J.L.); 2 Energy Storage Management and Equipment Group, Qingdao Institute of Bioenergy and Bioprocess Technology, Chinese Academy of Sciences, Qingdao 266101, Shandong, China

**Keywords:** sulfonated poly(ether ether ketone), ammonium ionic liquids, nano silicon dioxide, leaching, proton conductivity

## Abstract

Nano-silicon dioxide (SiO_2_) was incorporated into the matrix of sulfonated poly(ether ether ketone) (SPEEK)/ammonium ionic liquid (AIL) membranes by solution casting, with the aim of reducing ionic liquid leaching and any consequent decrease in proton conductivity. Fourier transform infrared (FTIR) spectra results indicate incorporation of the SPEEK matrix with AIL and nano-SiO_2_. Scanning electron microscopy (SEM) and X–ray diffraction (XRD) images revealed that incorporation of nano-SiO_2_ make the ternary composite membranes more flexible. The maximum ion exchange capacity (IEC) value was 3.25 meq/g, and the net structure formed by the nano-SiO_2_ not only retarded AIL leaching, but also increased the proton conductivity of the composite membranes. AIL leaching from the membranes was between 20% and 30%, which was lower than that of the SPEEK/AIL membrane. The maximum proton conductivity for the SPEEK/AIL/SiO_2_ membranes reached 10.73 mS/cm at 393 K.

## 1. Introduction

Fuel cells offer high power densities and energy conversion efficiencies, and also produce limited amounts of environmental pollution; they are thus considered to be clean energy sources that have the potential to replace fossil fuels [1]. Of the different fuel cell types, the polymer electrolyte membrane fuel cell (PEMFC) has attracted substantial attention because of its particular advantages, such as its rapid start–up at room temperature [2]. The polymer electrolyte membrane (PEM) is one of the critical components of the PEMFC, and it is used in proton conduction and to prevent fuel mixing. However, to date, only membranes based on perfluorosulfomic acid (PFSA), such as Nafion (Dupont) and GORE–SELECT (W.L. Gore and Associates. Inc.), have been considered competent enough for use in practical PEMFC applications, because of their good chemical and mechanical stability and high proton conductivity [3,4,5]. The PFSA membrane depends on water to transmit protons in the form of H_3_O^+^, but it has a high cost of $500–800 per m^−2^ and provides extremely low proton conductivity (0.5 mS cm^−1^) at high temperatures (>373 K) in relation to the evaporation of water. In contrast, the high-temperature PEMFC (HT–PEMFC) operates under low humidity and even anhydrous conditions. It also has many other merits: it tolerates impure fuel streams (with a CO tolerance of 1000 ppm at 403 K), employs an uncomplicated method to manage water and heat, and has quicker electrode reaction kinetics [6,7]. Therefore, a considerable amount of research has been conducted with the aim of developing a new type of low-cost PEM with high proton conductivity, for use in HT–PEMFCs. 

Many types of electrolytes have been prepared for HT–PEMFCs over the past decades [8,9,10,11,12]: modified PFSA membranes, blend polymer electrolyte membranes, polybenzimidazole (PBI)/H_3_PO_4_ membranes, and ionic liquids/polymer membranes. Of these, the sulfonated poly(ether ether ketone) (SPEEK) membrane has been identified as a reasonable replacement for the PFSA as a PEM, because of its unique tensile strength, low development cost, high chemical resistance, and thermal stability [13,14]. To further improve the proton conductivity of SPEEK, some researchers have incorporated inorganic oxides into the original membrane [15,16,17,18], including silicon dioxide (SiO_2_), TiO_2_, ZrO_2_, which have high melting points, good chemical stabilities, perfect moisture absorption properties, and provide excellent mechanical performances. Prior to use, these inorganic oxides are treated by modifying their surfaces or making them nano–sized to improve their specific surface area, which thus strengthens their ability to adsorb and desorb water molecules. For example, Mossayebi [15] found that SPEEK incorporated with sulfated zirconia nanoparticles had higher proton conductivity, oxidative stability, and water absorption capability, and the maximum proton conductivity of a SPEEK/sulfonated zirconia was found to reach 3.88 mS/cm at 100% relative humidity (RH) and 373 K. However, Li [16] prepared a cross–linking hybrid membrane incorporating 3%–8% SiO_2_, and observed a decrease in the proton conductivity, although the dimensional stability of the membrane improved when the mass fraction of SiO_2_ within it increased. Salarizadeh [17] incorporated amine-functionalized iron titanate (AIT) into a SPEEK polymer matrix, and the proton conductivity of the obtained SPEEK/AIT membrane was observed to reach 120 mS/cm at 353 K, in relation to the water channels formed at the interface between the polymer and nanoparticle; the connectivity of water channels provided an increase in the number of direct proton conduction routes. As ionic liquids (IL) possess certain merits, such as high proton conductivity, low viscosity, negligible vapor pressure, and good electrochemical and thermal stability [19], other researchers [20,21] have attempted to incorporate IL within the SPEEK matrix to enhance membrane proton conductivity. In this respect, the proton conductivity of a SPEEK/alkylimidazolium membrane reaches 3.16 mS/cm under anhydrous conditions at 418 K, and the highest power density of the PEMFC using the prepared membrane reaches 203 mW/cm^2^ at 418 K [21]. However, SPEEK/IL membranes undergo IL leaching when the PEMFC is running for a long time, which ultimately causes attenuation of membrane proton conductivity and fuel cell performance [20]. In our previous study [22], ammonium ionic liquid (AIL) was fabricated and incorporated within the SPEEK matrix to enhance compatibility between the ionic liquid and the Pt/C catalyst. However, neither proton conductivity nor the AIL loss rate of SPEEK/AIL membranes reached our expectations. Therefore, in this study, AIL and nano-SiO_2_ were incorporated into the matrix of SPEEK, with the aim of enhancing proton conductivity and AIL retention within the SPEEK/AIL membrane.

In this study, SPEEK/AIL/SiO_2_ ternary composite membranes were fabricated using a solution casting method. Ternary composite membrane morphology was characterized by Fourier transform infrared (FTIR), scanning electron microscopy (SEM), and X–ray diffraction (XRD). In addition, thermogravimetric analysis (TGA) was used to evaluate thermal stability. The electrochemical characteristics of the SPEEK/AIL/SiO_2_ composite membranes, such as IEC, proton conductivity, and AIL leaching, were also analyzed.

## 2. Experimental

### 2.1. Sulfonated Poly(ether ether ketone)/Ammonium Ionic Liquid/Silicon Dioxide Composite Membrane Preparation

SPEEK was manufactured using the direct sulfonation method. First, 5 g of poly(ether ketone) (PEEK; 450 PF, purchased from Victrex Inc., Houston, TX, USA) was dissolved in 95 mL sulfuric acid (98 wt %) and agitated vigorously for 3–12 h at 313–353 K [23]. The mixed solution was then added slowly to iced water under stirring. The precipitate was washed repeatedly with de–ionized water until a pH of 7 was attained, and it was then dried in a vacuum oven at 353 K for 20 h. The degree of sulfonation (DS) of the prepared SPEEK was detected using the ^1^H nuclear magnetic resonance (NMR) spectrum [24]. 

Neutralization was used to make the AIL [25]. H_2_SO_4_ was mixed with triethylamine at 333 K for 1 h under continual stirring, and the mixed solution was then heated to 343 K for 3 h. The reaction product was purified at 353 K in a rotary evaporator, until the quality of the remaining amount was unchanged. Ultimately, the prepared AIL was ammonium hydrogen sulfate ([(CH_3_CH_2_)_3_NH^+^] [HSO_4_^−^], TEAS) [22].

Nano-SiO_2_ was prepared using a sol–gel method. Tetraethyl orthosilicate (TEOS) was added to ethanol, and hydrochloric acid (HCl) was then added dropwise. The molar ratio of HCl:ethanol:TEOS:H_2_O was 10:6:1:30. The solution was mixed by magnetic stirring for 3 h at 333 K to form a colorless and transparent solution, and then dried in a vacuum oven at 333 K for 24 h to produce a colorless gel. When the gel had dried, it was crushed into a fine powder using a mortar, and then stored in a zip lock bag for future use.

**Figure 1:**
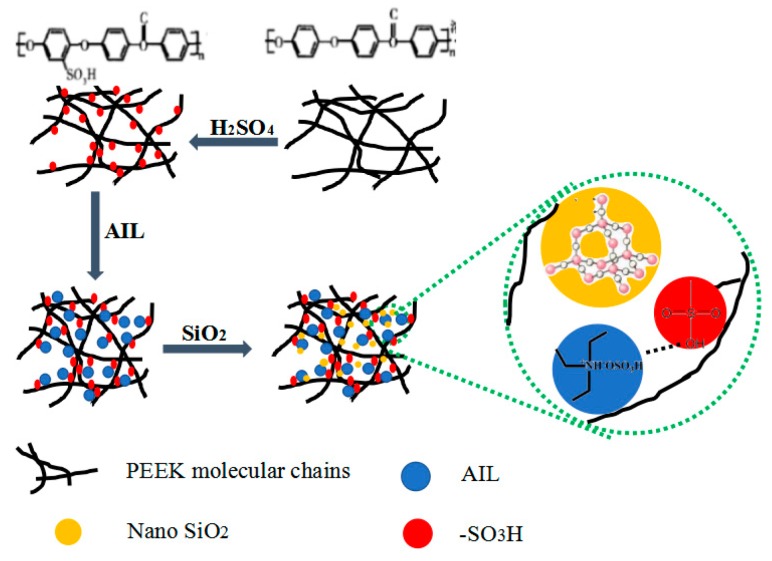
Schematic representation of the ternary composite membrane preparation process.

The SPEEK/AIL/SiO_2_ composite membranes were fabricated using a solution casting method. First, the nano-SiO_2_ powder and AIL were added into SPEEK/DMAC (10 wt %) solution under magnetic stirring for 3–5 h at 333 K. The mixed solution was then cast onto a polytetrafluoroethylene (PTFE) board and dried under vacuum at 333 K for 12 h, and then again at 363 K for 8 h, to obtain final SPEEK/AIL/SiO_2_ ternary composite membranes. The entire preparation process used to prepare the ternary composite membranes is shown in Figure 1. Please note the following abbreviations used in this article: the “X” within the symbol SPEEK–X/AIL/SiO_2_–Y denotes the DS of the SPEEK; AIL is the ammonium ionic liquid ([(CH_3_CH_2_)_3_NH^+^] [HSO_4_^−^], TEAS); “Y” is the mass fraction of nano-SiO_2_ for the total mass of the ternary composite membranes. In the prepared composite membranes, the AIL mass accounted for 35% of SPEEK used in all prepared composite membranes, since the PEMFC performance using the SPEEK75–35S membrane provided the best result in our previous work [22].

### 2.2. Characterization of Sulfonated Poly(ether ether ketone)/Ammonium Ionic Liquid/Silicon Dioxide Composite Membrane

#### 2.2.1. Fourier Transform Infrared Spectra 

FTIR spectra of PEEK, SPEEK, AIL, and SPEEK/AIL/SiO_2_ composite membranes were measured in a wave number range between 600 cm^−1^ and 4000 cm^−1^ using a Thermo Scientific Nicolet iS 10 (Thermo Fisher Scientific, Waltham, MA, USA). The spectra were measured in transmittance mode with a resolution of 2 cm^−1^.

#### 2.2.2. X–ray Diffraction

XRD patterns of SPEEK, SPEEK/AIL, and SPEEK/AIL/SiO_2_ ternary composite membranes were recorded using a D–MAX 2500 (Rigaku, Tokyo, Japan). The voltage and current of X–ray tubes were 40 KV and 150 mA, respectively, and XRD patterns were obtained at 2 θ, varying between 5° and 80°.

#### 2.2.3. Scanning Electron Microscopy

The morphology of composite membranes was observed using a Jeol scanning electron microscope (SEM, JSM–7800F, JEOS Ltd, Tokyo, Japan).

#### 2.2.4. Thermal Properties

To determine the thermal stability of the synthesized SPEEK/AIL/SiO_2_ ternary composite membranes, a thermogravimetric analysis (TGA) was conducted under a nitrogen gas atmosphere using a TG 209F1 TGA analyzer (TA instrument, Bochum, Germany) within a temperature range of 303 K to 873 K and at a heating rate of 10 ℃/min.

#### 2.2.5. Ion Exchange Capacity

The ion exchange capacity (IEC) of SPEEK/AIL/SiO_2_ membranes was measured using traditional acid–base titration methods. First, the mass of the ternary composite membrane sample was weighed using an analytical balance, and the sample was then immersed in 20 ml 2 mol/L NaCl solution for 24 h. The membrane sample was then extracted from the solution and washed, using de-ionized water to remove the residual NaCl. Phenolphthalein was added to the solution as an indicator, and the solution was titrated by NaOH solution (*M*_NaOH_ = 0.01 mol/L) until a faint pink color was obtained. The volume of the NaOH solution (*V*_NaOH_) was recorded. At least three measurements were taken for each membrane sample. The IEC value of the SPEEK/AIL/SiO_2_ composite membrane was obtained using Equation (1):

(1) $$IEC=\frac{V_{NaOH}\times M_{NaOH}}{weight\;of\;dry\;membrane}$$

#### 2.2.6. Proton Conductivity

The proton conductivity of the SPEEK/AIL and SPEEK/AIL/SiO_2_ membranes was measured using AC impedance spectroscopy in a frequency range of 100 Hz to 1 MHz and at a voltage amplitude of 10 mV, using an electrochemical workstation (CHI660E, Shanghai Chenhua instrument Co. Ltd., Shanghai, China). Each membrane sample was nipped between two carbon-paper electrodes, each with an area of 0.332 cm^2^. The thickness of the SPEEK/AIL/SiO_2_ composite membrane was 50 μm, and its proton conductivity value was calculated by applying Equation (2):

(2) $$\sigma =\frac{L}{R\cdot A}$$

where, *σ* refers to proton conductivity, *R* is membrane resistance, *L* is membrane thickness, and *A* is the contact area of the electrode. The membrane resistance (*R*) value of SPEEK/AIL/SiO_2_ composite membranes was acquired using a Nyquist curve by equivalent circuit, fitting with Z–view software.

#### 2.2.7. Ammonium Ionic Liquid Leaching of Sulfonated Poly(ether ether ketone)/Ammonium Ionic Liquid/ Silicon Dioxide membranes

SPEEK/AIL/SiO_2_ composite membranes were dried in a vacuum oven at 353 K until a constant mass was attained (W_1_ g), and then dipped in de–ionized water at 373 K for 0.5 h. The membrane samples were then dried again in a vacuum oven at 353 K until a constant mass (W_2_ g) was attained, and then re-dipped in de-ionized water for 1 h, dried, and re–weighed (W_3_ g). The weight of the dry samples was measured in the same way after immersion in water for 2 h and 4 h. AIL leaching of the SPEEK/AIL/SiO_2_ composite membranes was then calculated using Equation (3):

(3) $$\mathrm{AIL}\;\mathrm{leaching}\;(\%)=\frac{W_{n}-W_{n+1}}{W_{n}}$$

## 3. Results and Discussion

### 3.1. Fourier Transform Infrared Spectra of the Membrane 

**Figure 2:**
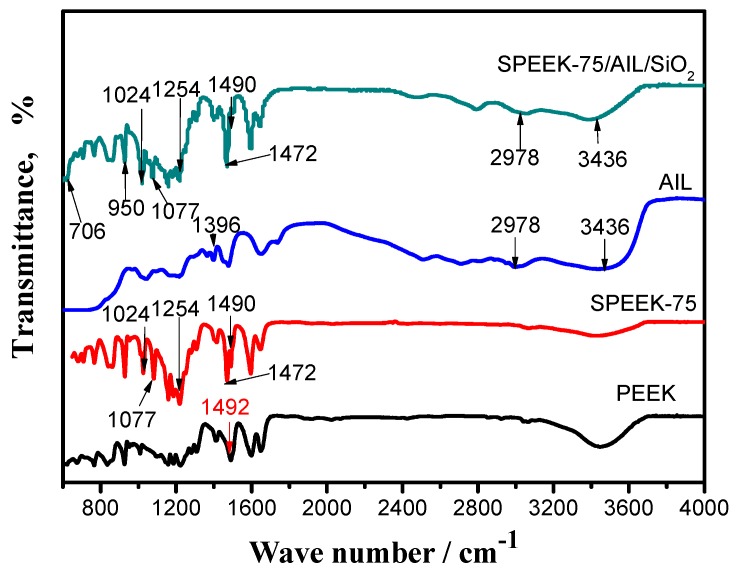
The Fourier transform infrared (FTIR) spectra of the poly(ether ether ketone) (PEEK), sulfonated poly(ether ether ketone) (SPEEK)–75, AIL, and SPEEK–75/AIL/SiO_2_ composite membranes.

The chemical structures of PEEK, SPEEK–75, AIL, and SPEEK–75/AIL/SiO_2_ composite membranes were qualitatively analyzed using FTIR spectra, as shown in Figure 2. The absorption peak of pristine PEEK was confirmed at 1492 cm^−1^, and this demonstrates the existence of a C–C aromatic ring [26], although the peak identity was reduced. The absorption of SPEEK was divided into two peaks, at 1490 cm^−1^ and 1472 cm^−1^ [27,28]. Compared to pristine PEEK, the new absorption peaks of SPEEK were at 1024 cm^−1^, 1077 cm^−1^, and 1254 cm^−1^, which reflect the existence of sulfonic acid groups within the SPEEK matrix. The three characteristic absorption peaks of the sulfonyl groups can be attributed to asymmetric O=S=O stretching, symmetric O=S=O stretching, and S=O stretching, respectively [28,29]. The characteristic peak of AIL at 3436 cm^−1^ shows –NH stretching vibration. Nitrogen atoms have strong electronegativity, and hydrogen bonds are formed at 3436 cm^−1^. The peak at 2978 cm^−1^ represents the stretching vibration absorption of –CH_3_, and 1396 cm^−1^ represents the bending absorption of –CH_2_ when connecting with the positively charged nitrogen center [25]. Characteristic peaks of SPEEK and AIL were also observed in the FTIR spectra of the SPEEK–75/AIL/SiO_2_ composite membrane samples, which demonstrates the presence of SPEEK and AIL within the ternary composite membrane. Furthermore, the absorption peaks at 950 cm^−1^ and 706 cm^−1^ accorded with the characteristic absorption peaks of silica, which are ascribed to the linear and bending stretching of Si–O–Si, respectively [30]. An asymmetric stretching vibration peak of Si–O–Si at 1075 cm^−1^ was anticipated; however, this peak was found to overlap with the peak associated with the symmetric stretching of the O=S=O of sulfonyl group at 1077 cm^−1^, although the absorption peak was much smaller than that of the SPEEK sample. These FTIR spectra results indicate that the SPEEK matrix had been incorporated with AIL and SiO_2_. In other words, the results indicate completion of SPEEK/AIL/SiO_2_ ternary composite membrane preparation.

### 3.2. Microstructure of Membranes

**Figure 3:**
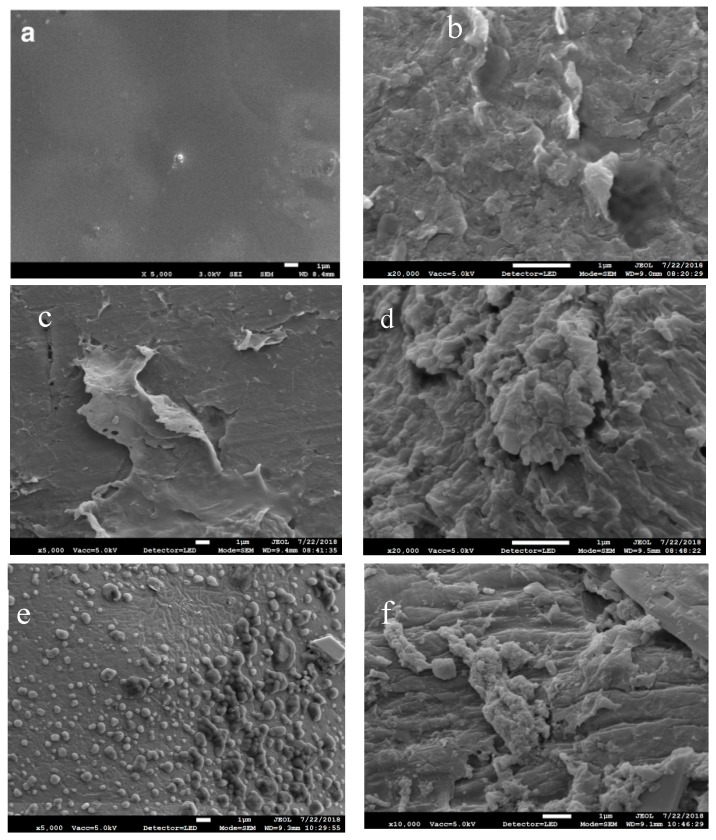
Scanning electron microscopy (SEM) images of membranes: (**a**) SPEEK–75/AIL; (**b**) SPEEK–75/AIL/SiO_2_–5; (**c**) SPEEK–75/AIL/SiO_2_–8; (**d**) SPEEK–75/AIL/SiO_2_–10; (**e**) SPEEK–75/AIL/SiO_2_–13; (**f**) SPEEK–75/AIL/SiO_2_–17.

SPEEK consists of a hydrophobic aromatic backbone and a hydrophilic side chain (terminated with sulfonic acid groups), and it has distinct nanophase separation. The aromatic backbone is formed in a hydrophobic phase and provides mechanical strength, whereas the side chain is aggregated into ionic clusters [31]. The morphologies of the prepared membranes were explored by SEM and XRD. Figure 3 shows SEM images of SPEEK–75/AIL and SPEEK–75/AIL/SiO_2_ membranes with different nano-SiO_2_ contents. It is clear from the SEM images in Figure 3a that SPEEK–75/AIL membranes have a smooth uniform morphology, with no obvious defects. The ternary composite membrane shown in Figure 3b–f shows that nano-SiO_2_ particles are entrapped within the continuous matrix, which suggests that the SPEEK network surrounding the nano-SiO_2_ particles forms a strong backbone, leading to flexibility of the ternary composite membrane. 

**Figure 4:**
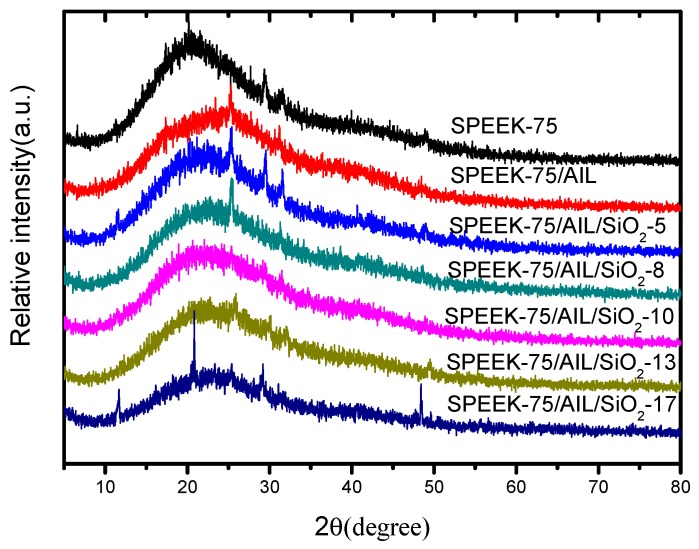
X-ray diffraction (XRD) patterns of the SPEEK/AIL/SiO_2_ composite membranes.

XRD was used to further investigate the influence of AIL and SiO_2_ on the morphology of the SPEEK matrix, and Figure 4 shows the XRD patterns of SPEEK–75, SPEEK–75/AIL, and SPEEK–75/AIL/SiO_2_ membranes with different nano-SiO_2_ contents. The XRD profiles show that all membranes have a broad crystalline band at 2 θ equaling 12°–30°, which corresponds to the ordered stacking of the hydrophobic backbone [32]. Compared to the SPEEK membrane, the intensity decline of this band in the SPEEK–75/AIL membrane can be attributed to the plasticizing effect of AIL on the hydrophobic domains of SPEEK. The plasticizing effect weakens the interaction between backbones and thus destroys the ordered stacking, thereby rendering the SPEEK–75/AIL membrane more flexible [33]. The crystallinity of the ternary composite membrane decreased with an increase in the nano-SiO_2_ content, from 5% to 17%. These results indicate that the amorphous domain is greatly fortified in the ternary composite membrane.

### 3.3. Thermogravimetric Analysis

**Figure 5:**
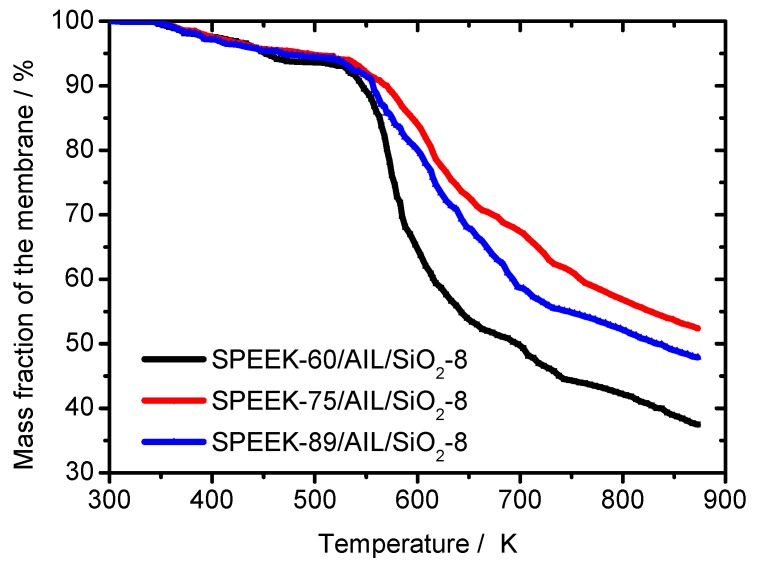
Thermogravimetric analysis (TGA) of the SPEEK/AIL/SiO_2_ composite membranes.

TGA was used to study the thermal stability of the SPEEK/AIL/SiO_2_ composite membranes, with respect to their potential use in HT–PEMFC applications. Figure 5 shows the mass loss of SPEEK/AIL/SiO_2_ composite membranes in a temperature range from 303 K to 873 K, and three mass loss stages are evident within this range. The first stage (350–373 K) represents the evaporation of absorbed water and residual solvent within the composite membranes [17]; the second stage (523–723 K) represents decomposition of the sulfonic acid group of SPEEK [29], AIL, and hydroxyl components adsorbed on the SiO_2_ sol; and the third stage (>723 K) represents decomposition of the SPEEK polymer chain. The thermal stability of the SPEEK–75/AIL/SiO_2_–8 composite membrane is superior to that of the other two membranes, and thus this membrane is suitable for use with HT– PEMFC.

### 3.4. Ion Exchange Capacity

**Figure 6:**
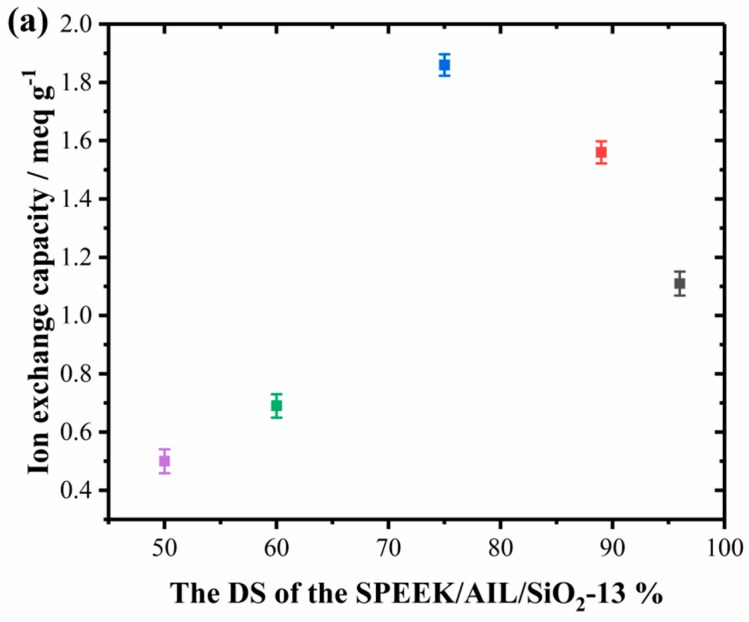

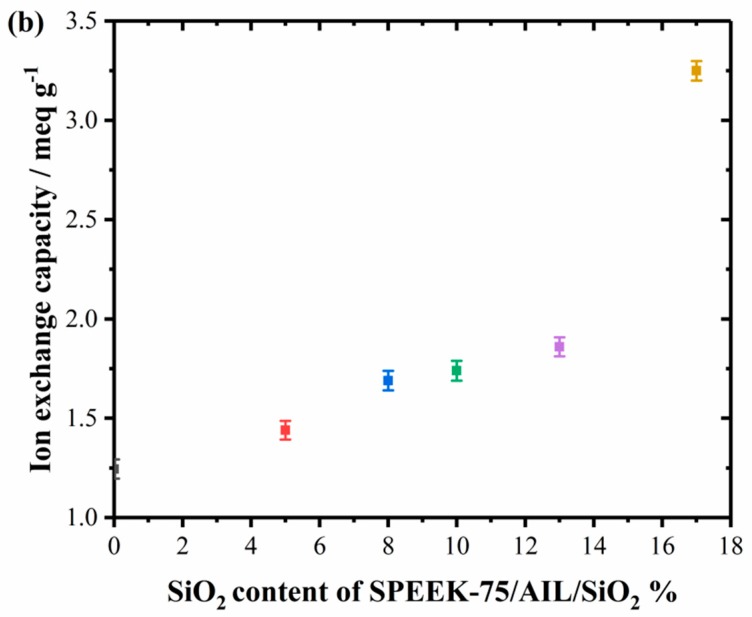
Ion exchange capacity (IEC) of the SPEEK/AIL/SiO_2_ composite membranes with different degree of sulfonation (DS) (**a**) and SiO_2_ content (**b**).

The IEC is usually defined as the number of sulfonic acid group per gram of the sulfonated polymer membrane, and it can be used to indicate sulfonic acid groups present within the polymer matrix that facilitate proton transfer. It also offers a credible approximation of membrane proton conductivity. The IEC value of SPEEK/AIL/SiO_2_ composite membranes with different DS and SiO_2_ contents are provided in Figure 6, where it is evident that the IEC values of the SPEEK/AIL/SiO_2_ composite membranes have a tendency to increase with an increase in the SiO_2_ content, and tend to increase and then decrease with an increase in DS. For example, the IEC value increased from 1.2 to 3.25 meq/g with an increase in the SiO_2_ content within the membrane, from 0% to 17%, while the IEC value first increased from 0.5 to 1.86 meq/g with an increase in the DS of the membrane from 50 to 75, but then decreased from 1.86 to 1.11 meq/g with an increase in DS from 75 to 96. These tendencies were further confirmed by the results of proton conductivity for the SPEEK/AIL/SiO_2_ membranes, as shown in the following section. Compared to the SPEEK/AIL membrane, the ternary composite membranes show enhanced IEC values. Generally speaking, the higher IEC values are attributed to the larger number of acid groups present in the SPEEK/AIL/SiO_2_ ternary composite membrane. This enhanced acidic property is also expected to improve membrane proton conductivity [34]. 

### 3.5. Proton Conductivity

**Figure 7:**
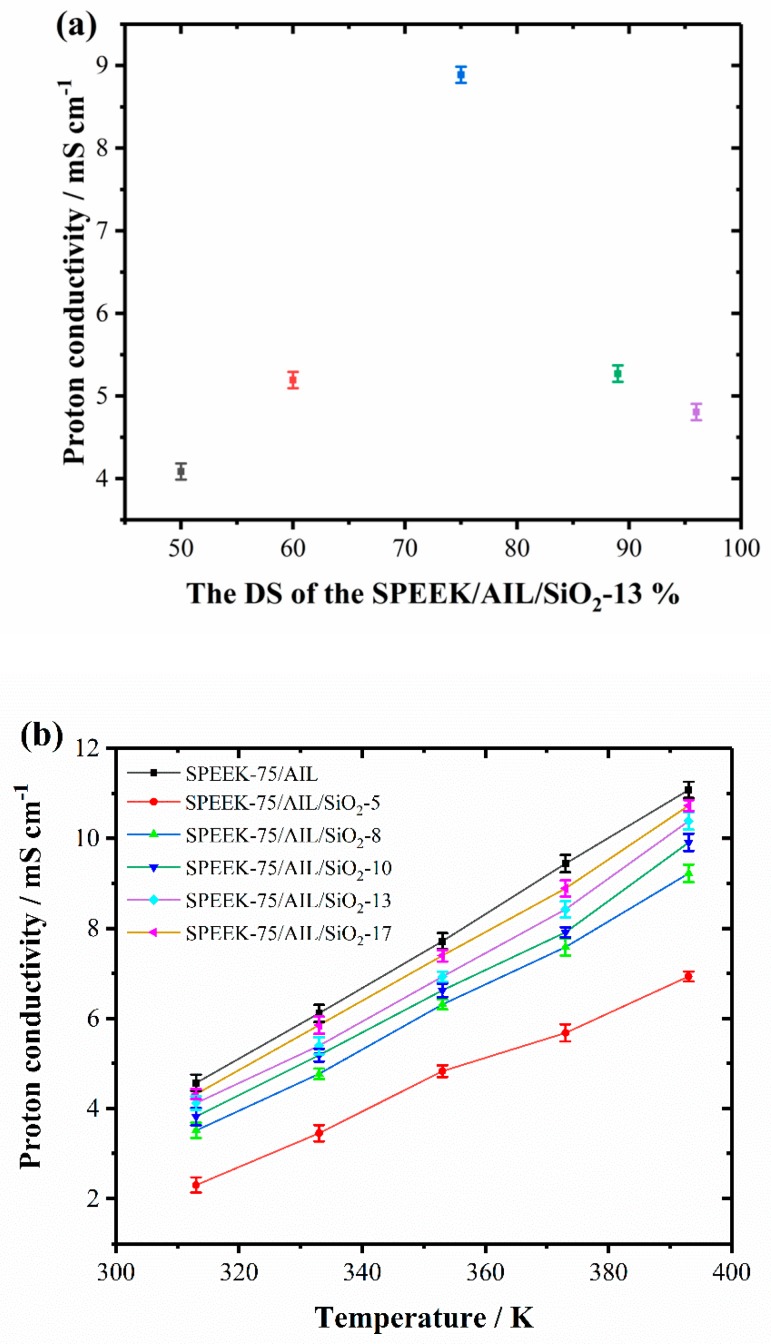
The proton conductivity of SPEEK/AIL/SiO_2_ membranes with different DSs (**a**) and SiO_2_ content (**b**).

The proton conductivities of the SPEEK/AIL/SiO_2_ composite membrane with different DSs at 373 K and different SiO_2_ contents at different temperatures are shown in Figure 7a,b. A maximum proton conductivity of 8.889 mS cm^−1^ is evident in Figure 7a, which occurs with a DS of 75 at 373 K in the ternary composite membrane. With an increase in the DS, the proton conductivity of the composite membrane followed the same tendency as the IEC values. The ammonium ionic liquid in the SPEEK/AIL/SiO_2_ composite membrane not only provides abundant water-free jumping points, but also forms an acid–base pair (SO_3_H–NH_2_) along the channel surface. The acid–base pairs offer new pathways for intensifying proton transfer and thus increase the proton conductivity of the composite membrane. As shown in Figure 7b, the proton conductivity of the SPEEK–75/AIL/SiO_2_ membrane was observed to increase with an increase in the SiO_2_ content within the composite membrane, which was expected. The nano-SiO_2_ particles within the composite membrane have a spatial net structure that not only stores water, but also anchors the AIL within the polymer matrix to prevent AIL leaching. Moreover, the surface of SiO_2_ contains a large number of hydroxyl groups, and also increases the water content in the composite membrane. With an increase in the SiO_2_ content, the cross-linking degree of the membrane was enhanced. In addition, the ability of the membrane to retain water and AIL increased, and the number of proton carrier sites also increased, leading to enhancement of membrane proton conductivity. The proton conductivity of the SPEEK–75/AIL/SiO_2_–17 composite membrane reached 10.73 mS cm^−1^ at 393 K. Figure 7b also reveals that the proton conductivity of all the prepared membranes with different SiO_2_ contents monotonically increased with an increase in temperature. For example, the proton conductivity of SPEEK–75/AIL/SiO_2_–17 increased from 4.322 to 10.73 mS cm^−1^ with a temperature increase from 313 K to 393 K. 

To further study the influence of the SiO_2_ content on the proton conductivity of the SPEEK/AIL/SiO_2_ composite membrane, the activation energy (*Ea*) for proton conduction through the membrane was calculated using Equation (4):

(4) $$\sigma =\sigma_{0}\exp(\frac{-E_{a}}{RT})$$

where *σ*_0_ is the pre–exponential factor, *R* is the ideal gas constant (8.314 J mol^−1^ K^−1^), *T* is the thermodynamic temperature, and *E*_a_ is activation energy for proton transfer. The value of *E*_a_ was estimated from the slope of a linear fitted curve of log *σ* versus 1000/T, and the results are shown in Table 1. The results were approximately equal to those reported in literature [35], and the SPEEK membrane reached a Grotthuss-type *E*_a_ of 14.0 kJ mol^−1^. In contrast, the incorporation of nano-SiO_2_ caused the *E*_a_ value of the SPEEK/AIL composite membrane to reduce to 12.3, 11.9, 11.7, 11.5, and 11.3 kJ mol^−1^ with an SiO_2_ doping content of 5%, 8%, 10%, 13%, and 17%, respectively. In conclusion, the reduced *E*_a_ and improved proton conductivity further evidence the conductive ability of the ammonium-type ionic liquids within the polymer electrolyte membrane. The conductive ability is improved by the existence of channels allowing Grotthuss-type proton transport, which is achieved through the incorporation of nano-SiO_2_ into the SPEEK/AIL membrane matrix. 

### 3.6. Ammonium Ionic Liquid Leaching

**Figure 8:**
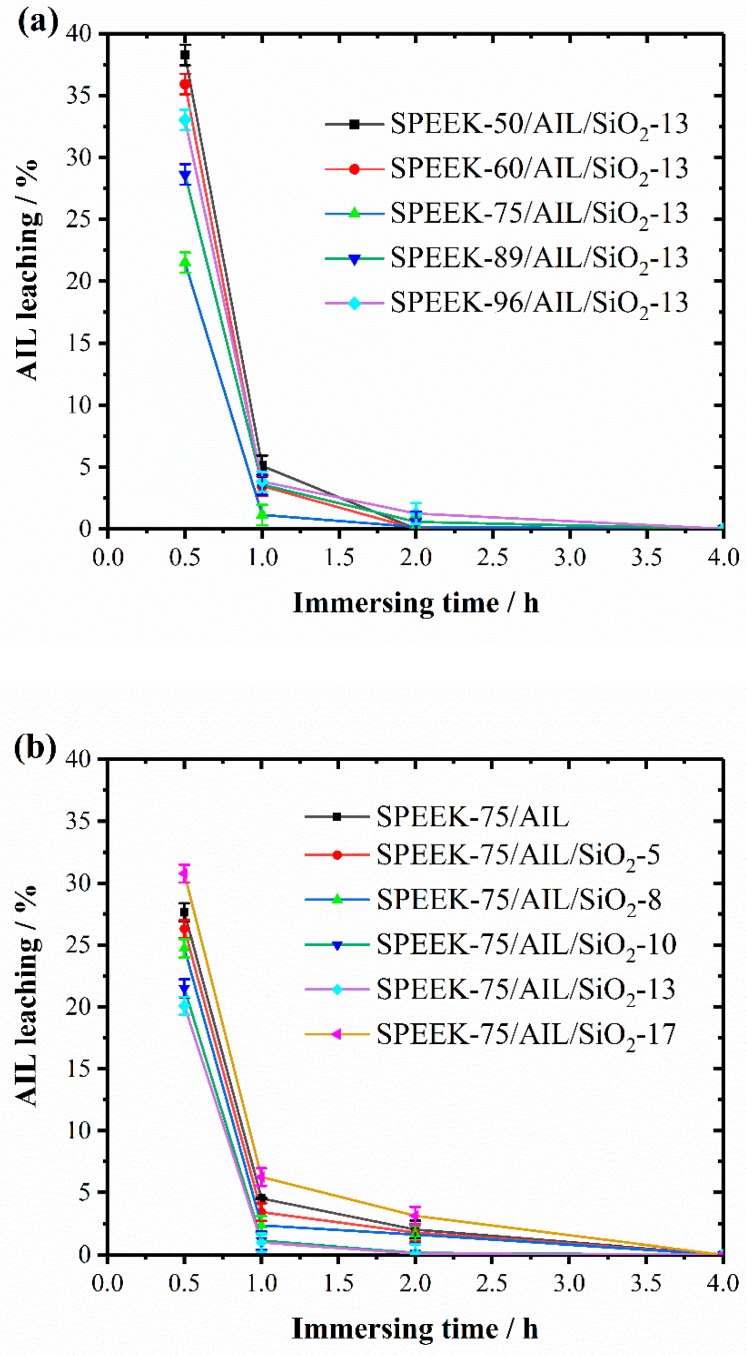
The AIL leaching of the SPEEK/AIL/SiO_2_ composite membranes with different DSs (**a**) and SiO_2_ content (**b**) as a function of time.

Generally speaking, IL leaching of the membrane is a problem for the IL-doped electrolyte membrane, as it reduces proton conductivity and thus decreases the performance of the fuel cell. During HT–PEMFC operation, water molecules are yielded at the cathode in the form of steam, and it is believed that some of these caused AIL leaching from the composite membrane. To investigate the retention of AIL within the SPEEK/AIL/SiO_2_ membrane, AIL loss was measured under extreme conditions (immersing the membrane sample into de-ionized water). Figure 8a,b show AIL leaching of SPEEK/AIL/SiO_2_ composite membrane samples with different DS and SiO_2_ contents as a function of time when dipped in de–ionized water. It is evident that all membrane samples show quick AIL loss in the initial hour from free-form AIL leaching [33]. Free-form AIL has a loose connection with the membrane substrate, and is mostly situated at the surface of the SPEEK/AIL/SiO_2_ composite membrane following physical adsorption. After a period of 1 h, the AIL content within the ternary composite membranes remained unchanged with an increase in the measuring time, which showed that residual AIL was in a bound form. AIL in bound form has strong connections with –SO_3_H and SiO_2_ groups within the composite membrane. AIL leaching of the SPEEK/AIL/SiO_2_ composite membrane was between 20% and 30%, which was lower than that of the SPEEK/AIL membrane. The nano-SiO_2_ net structure and SPEEK twist strengthened the tightness of the net structure, which effectively reduced AIL leaching from the SPEEK/AIL/SiO_2_ composite membrane. 

## 4. Conclusions

SPEEK/AIL/SiO_2_ ternary composite membranes were fabricated using a solution casting method. FTIR spectra results indicated that the SPEEK matrix had been incorporated with AIL and SiO_2_. SEM and XRD images showed that the incorporation of nano-SiO_2_ rendered the ternary composite membrane more flexible. Furthermore, TGA curves of the prepared ternary composite membranes indicated that the membranes were appropriate for use in HT–PEMFC. The IEC value of the SPEEK/AIL/SiO_2_ composite membranes showed a tendency to increase with an increase in the SiO_2_ content, but a tendency to increase and then decrease with an increase in the DS. Higher IEC values were attributed to the presence of a greater number of acid groups within the ternary polymer electrolyte membrane. With an increase in the SiO_2_ within the ternary composite membrane, there was an enhancement of the cross–linking degree of the membrane, which improved the membrane’s AIL retention ability and proton conductivity. AIL leaching from SPEEK/AIL/SiO_2_ membranes was between 20% and 30%, which was lower than that of SPEEK/AIL membranes. In this paper, incorporation of nano-SiO_2_ into the SPEEK/AIL matrix raised proton conductivity and the AIL retention of the membranes, and the membranes therefore have potential application for use in HT–PEMFC.

## Figures and Tables

**Table 1:** The activation energy for proton conduction through SPEEK/AIL/SiO_2_ composite membranes.

Membrane	Activation Energy (kJ mol^−1^)
SPEEK–75/AIL	14.0
SPEEK–75/AIL/SiO_2_–5	12.3
SPEEK–75/AIL/SiO_2_–8	11.9
SPEEK–75/AIL/SiO_2_–10	11.7
SPEEK–75/AIL/SiO_2_–13	11.5
SPEEK–75/AIL/SiO_2_–17	11.3
